# Automated Coffee Roast Level Classification Using Machine Learning and Deep Learning Models

**DOI:** 10.1111/1750-3841.70532

**Published:** 2025-09-09

**Authors:** René Ernesto García Rivas, Pedro Luiz Lima Bertarini, Henrique Fernandes

**Affiliations:** ^1^ Faculty of Computing Federal University of Uberlandia Uberlândia Brazil; ^2^ Faculty of Electrical Engineering Federal University of Uberlandia Uberlândia Brazil; ^3^ IVHM Centre, Faculty of Engineering and Applied Sciences Cranfield University Cranfield UK

## Abstract

The coffee roasting process is a critical factor in determining the final quality of the beverage, influencing its flavour, aroma, and acidity. Traditionally, roast‐level classification has relied on manual inspection, which is time‐consuming, subjective, and prone to inconsistencies. However, advancements in machine learning (ML) and computer vision, particularly convolutional neural networks (CNNs), have shown great promise in automating and improving the accuracy of this process. This study evaluates multiple ML models for coffee roast level classification, including a CNN with Xception as a feature extractor, alongside AdaBoost, random forest (RF), and support vector machine (SVM). The models were trained and tested on a public dataset of 1,600 high‐quality images, balanced across four roast levels: green, light, medium, and dark, to ensure robust performance. Experimental results demonstrate that all models achieved 100 % accuracy and F‐1 scores, confirming their effectiveness in accurately distinguishing roast levels. Furthermore, the proposed approach was compared with previous studies, showing strong performance in roast classification. Image augmentation techniques were applied to improve generalizability in real‐world applications. This research presents a reliable, scalable, and fully automated solution for roast‐level classification, significantly contributing to quality control in the coffee industry.

**Practical Applications:**

This research offers a reliable and automated way to classify coffee bean roast levels using image analysis and ML. It can help coffee producers and roasters improve quality control by providing faster, more consistent, and objective assessments of roast levels, ultimately ensuring a better product for consumers.

## 1 Introduction

Coffee roasting is a critical stage in coffee production, significantly influencing the final quality of the beverage. Proper roasting enhances key attributes such as flavour, aroma, and acidity, making it essential for delivering a high‐quality product (Motta et al. 2025). However, manually assessing the roast level presents several challenges, even for experienced professionals. The traditional methods are time‐consuming, subjective, and prone to inconsistencies, limiting rapid decision‐making and large‐scale evaluation (Patrício and Rieder 2018).

Accurate determination of roast levels is crucial to ensuring consistency and meeting consumer preferences. Historically, this task has relied on manual inspection and subjective judgment, which can introduce variability and errors (Chiang et al. 2018). However, advancements in ML, particularly CNNs, offer promising solutions for automating and improving the precision of this process. CNNs are well‐suited for complex pattern recognition tasks, making them ideal for analyzing the subtle colour variations and texture changes that occur during roasting (Dos Santos et al. 2020).

A comprehensive review of the literature highlights the effectiveness of CNNs in various coffee‐related applications, including bean maturity classification, defect detection, and quality control during roasting (Wallelign et al. 2019; Pimenta et al. 2018; García et al. 2019; Alessandrini et al. 2010; Metha et al. 2024; Rivalto et al. 2020; Arboleda et al. 2019). Within the roasting domain, several studies have focused on classifying roast levels using different datasets and ML models, including SVM, ResNet‐152, MobileNetV2, Fully Connected Neural Networks, SPSO, and DenseNet121 (Motta et al. 2025). Notably, (Septiarini et al. 2022) achieved high accuracy in classifying three roast levels using SVM, while (Janandi and Cenggoro 2020; Hakim et al. 2020) developed a mobile application for automatic roast degree classification.

Building on this foundation, our study aims to classify four roast levels (green, light, medium, and dark), evaluating a CNN with Xception as a feature extractor and multiple ML methods such as AdaBoost, RF, and SVM, comparing their performance to previous studies.

## 2 Literature Review

Coffee producers need to maintain consistent quality in their products. Traditional quality control methods are labour‐intensive and prone to human error (Pimenta et al. 2018).

According to (Dos Santos et al. 2020), automating the process with CNNs can save time and reduce costs. It can help identify and sort beans based on quality, ensuring that only the best beans are selected for premium products (Pereira Neto et al. 2023). CNNs can detect defects or irregularities in coffee beans, such as mould, insect damage, or other imperfections, which are critical for quality assurance. Automated systems can quickly handle large volumes of beans, making it easier to scale up operations without a proportional increase in labour (García et al. 2019).

Some of the ML methods used are: SVM, k‐nearest neural network (KNN), Probabilistic Neural Network (PNN), artificial neural network (ANN), multi‐layer perceptron (MLP), deep belief network (DBN), back‐propagation neural network (BPNM), principal component analysis (PCA), imperialist competitive algorithm (ICA), neural network intensity (NNI), and partial least squares discriminant analysis (PLS‐DA).

SVM is the most frequently used classification method, demonstrating its robustness in tasks such as disease detection and phenotyping (Phillips and Abdulla 2021; Dhakshina Kumar et al. 2020). ANN, DBN, and BPNN have also shown high accuracy, especially in tasks like fungal recognition, wheat purity classification, and germinated grain identification (Ebrahimi et al. 2014; Lüy et al. 2023).

**FIGURE 1:**
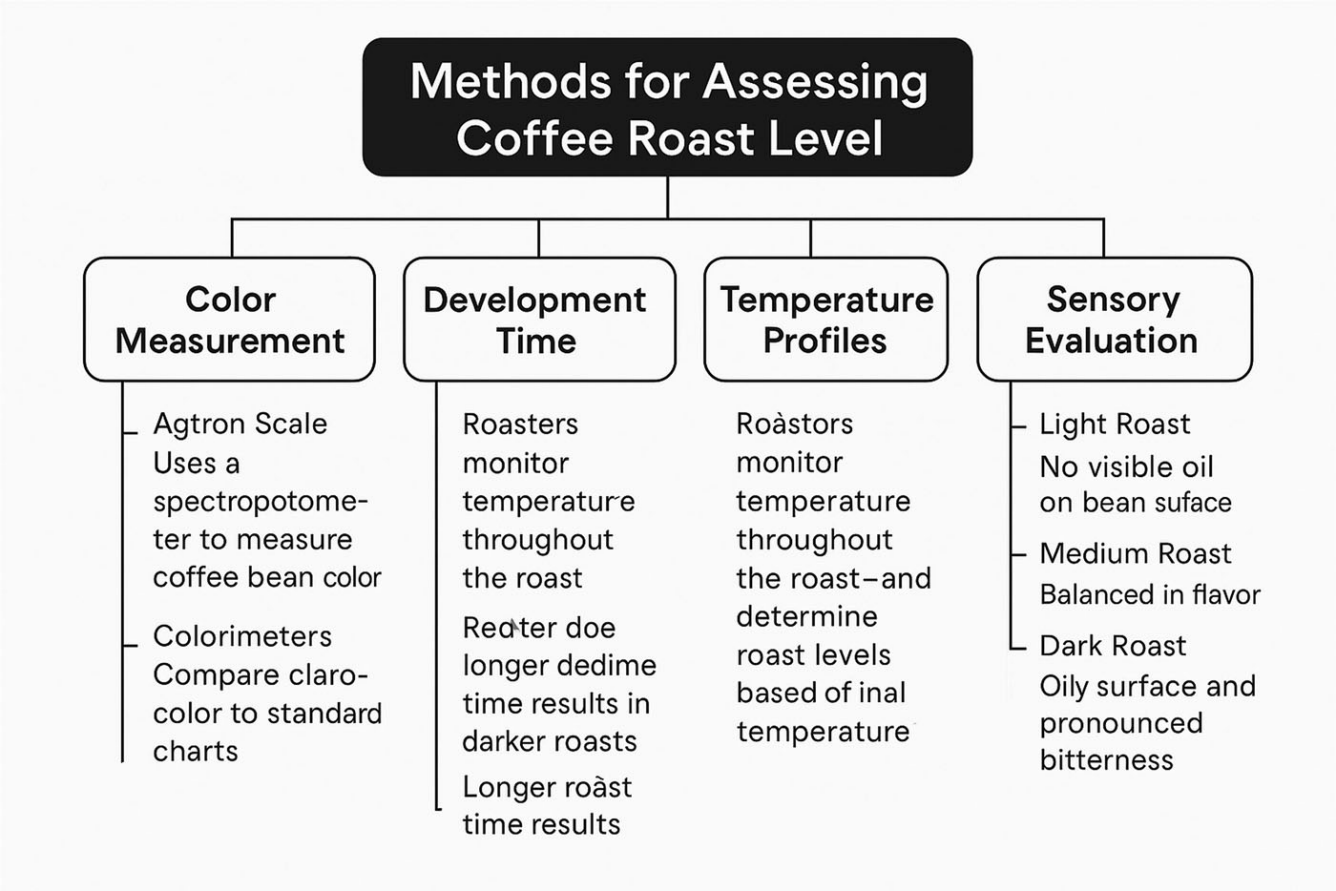
Methods for assessing coffee roast levels.

The roast level of coffee beans plays a vital role in shaping the final cup's flavour, aroma, and overall character (Arboleda et al. 2019;). Coffee roasting is both a science and an art, and professionals use various methods to assess how far the beans have been roasted, each offering unique insights into the process (Alessandrini et al. 2010). Some of the most widely used approaches include analyzing colour, tracking development time, monitoring temperature changes, and using sensory cues such as smell and sound (Metha et al. 2024). Traditional methods for assessing roast levels have several limitations, including subjectivity and inconsistency (Oliveira et al. 2023). Recent advances in ML and computer vision offer promising solutions to these challenges (Motta et al. 2025). In Figure 1, it is described different methods for assessing coffee roast levels.

**FIGURE 2:**
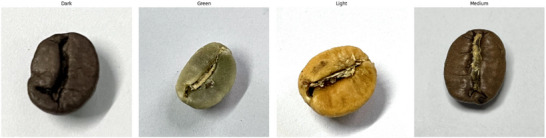
Types of coffee roast levels: dark, green, light and medium.

By using these methods, roasters can accurately determine and replicate the desired roast level, ensuring consistency in the flavour and quality of the coffee.

The pigmentation of coffee beans undergoes alteration as the thermal conditions escalate, resulting in classifications of roasts that may be categorized as light, medium, or dark with examples shown in Figure 2. Each classification is associated with distinct flavour profiles.

The roasting process is characterized by the temporal extent during which the coffee bean undergoes exposure to heightened thermal conditions under the scrupulous supervision of a qualified specialist. Investigations that elucidate the roasting continuum and analyze the operational dynamics until the coffee reaches its definitive carbonization stage are instrumental in promoting the automation of the procedure, consequently diminishing the reliance on specialized oversight. For instance, computer vision technologies are employed to observe the roasting procedure of coffee beans, emphasizing the variations in characteristics across different roasting levels (Bagdonaite and Murkovic 2018; Summa et al. 2007;).

As illustrated in Table 1, the first column corresponds to the reference of the literature review; the second column describes the paper's primary objectives; the third column lists the models and algorithms used in the paper; and the last column informs the results achieved. The models tested for coffee roast classification were SVM, ResNet‐152, MobileNetV2, fully connected neural network, SPSO, DenseNet121, and CNN proposed by (Motta et al. 2025). The research by (Septiarini et al. 2022) achieved maximum accuracy in classifying three roast levels using SVM.

**TABLE 1:** Papers related to coffee roast classification.

Source	Classification of the roasting level	Methodology	Best results achieved
(Metha et al. 2024)	Into four classes.	MobileNetV2 and VGG19. And a dataset of 1200 images was used.	Accuracy of 94.79% achieved by MobileNetV2 architecture.
(Arboleda et al. 2019)	Into three classes.	RGB values as the input in ANN.	Accuracy of 97.22%.
(Septiarini et al. 2022)	Into three classes.	Feature extraction and SVM method. A dataset of 150 images was used.	The polynomial kernel achieved a maximum accuracy of 100%.
(Janandi and Cenggoro 2020)	Into three classes: good, medium, and bad.	A dataset of 160 images was used. The proposed model was tested with ResNet‐152 and VGG16.	The best model was ResNet‐152, which achieved an accuracy of 73.3%.
(Hakim et al. 2020)	Into three classes: accepted, rejected, and not yet.	Four architectures and a dataset of 10,944 images.	The best model was MobileNetV2, which achieved an accuracy of 97.75%.
(Okamura et al. 2021)	Recognition of the brightness of the beans before and after grinding.	Five algorithms: linear regression, DT, RF, SVR, and a CNN.	The CNN performed the best, with a 2.52 colour numerical difference.
(Ratanasanya et al. 2022)	Optimal coffee bean roasting conditions.	Starling particle swarm optimization (SPSO).	Average errors of 1.2 to 8.5%.
(Bipin Nair et al. 2023)	Into seven different classes.	DenseNet121 architecture. And a dataset of 363 images was used	An accuracy of 81.89%.
(Vilcamiza et al. 2022)	Into three classes: under‐roasting, optimum‐roasting, and over‐roasting.	CNN using NVIDIA Jetson Nano and a dataset of 2489 images.	An accuracy of 91.33 %.
(N. K. Naik and Sethy 2022)	Into four classes.	CNN and a dataset of 1200 images.	An accuracy of 97.5%.
(Leme et al. 2019)	Into eight roasting levels.	The study develops a model for whole beans and a model for ground beans. A dataset of 165 samples was used.	The model for whole beans achieved a root‐mean‐square error of 0.99.
(Heide et al. 2020)	Prediction of the roast degree and the antioxidant capacity of the coffee brew.	Online single‐photon ionization time‐of‐flight mass spectrometry (SPI‐TOFMS) with a 5 s time resolution to analyze the chemical composition of the roasting off‐gas.	The model successfully predicted the roast degree and antioxidant capacity with root‐mean‐square errors of 6.0 and 139 mg of gallic acid equivalents per litre, respectively.

*Source*: the authors

In (Metha et al. 2024), using the same dataset, they used two architectures, namely VGG19 and MobileNetV2. They discussed how the model flows to obtain accuracy and validation values using MobileNetV2 and VGG19. MobileNet is usually designed specifically for mobile applications with limited resources or capacity. Mobilenet's priorities are speed and efficiency, which is suitable for mobile or edge computing devices. Meanwhile, VGG has a highly complex convolutional layer structure with more parameters than MobilenetV2.

In (Arboleda et al. 2019), authors used an ANN to classify the coffee beans' degree of roast into light, medium, and very dark roasts using the RGB values as the input in an artificial neural network. The result showed that the proposed method could accurately identify the coffee beans' degree of roasting with 97.22%.

## 3 Methodology

### 3.1 Methodological Framework for Coffee Beans Roast Level Classification

**FIGURE 3:**
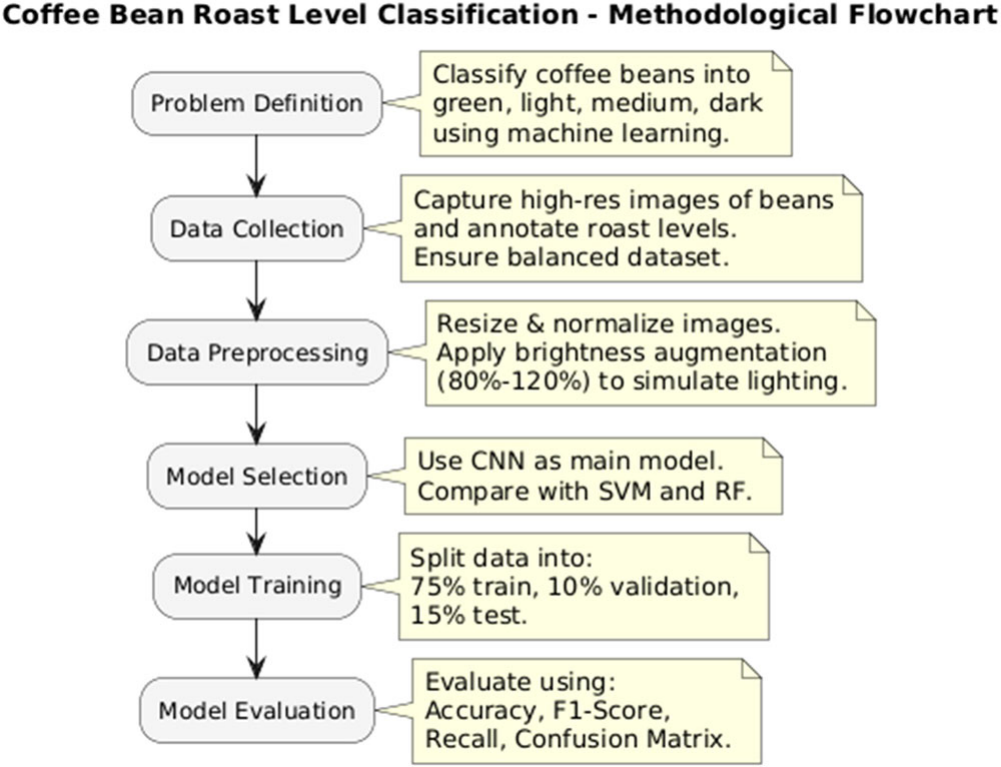
Methodological flowchart.

The methodology involves classifying coffee beans by roast level using image analysis and ML. Images labeled as green, light, medium, or dark are preprocessed through normalization, resizing, and brightness augmentation. A CNN is used alongside SVM andRF for comparison, Also the model performance is evaluated using accuracy, F1‐score, recall, and confusion matrix. These steps are summarized in Figure 3.

#### 3.1.1 Problem Definition

The central aim of this study is to classify coffee beans into four distinct roast levels—green, light, medium, and dark—using computer vision and ML techniques. This task is important for improving the consistency and quality control in coffee production, as roast level greatly influences flavor, aroma, and acidity. The challenge lies in developing a reliable automated system that can perform this classification based on images of the beans.

#### 3.1.2 Data Collection

The dataset in this study consists of 1600 images of roasted coffee beans, and it is available online (https://www.kaggle.com/datasets/gpiosenka/coffee‐bean‐dataset‐resized‐224‐x‐224) The dataset used is a resized version of the “Coffee Bean Dataset Version 1.” The coffee beans, sourced from Bona Coffee, consist of four roast levels: un‐roasted (Green) Laos Typica Bolaven, lightly roasted Laos Typica Bolaven, medium‐roasted Doi Chaang, and dark‐roasted Brazil Cerrado, all of which are Coffea Arabica. All photographs were captured using an iPhone 12 Mini with its 12‐megapixel back camera system. To ensure robustness and validate the model against a wide range of inputs, the imaging conditions were intentionally varied. Images were captured using both controlled LED lighting from a lightbox and ambient natural light. For each photograph, the camera was positioned to maintain a consistent, parallel plane to the coffee beans, which were placed in a container. The original images were saved in PNG format with a resolution of 3024 × 3032 pixels, later they were resized to 224 × 224 pixels (Ontoum et al. 2022).

The study utilizes a balanced dataset comprising 4800 images, which are evenly distributed across the four classification categories: green, light, medium, and dark (1200 images per class). To facilitate the ML pipeline, this dataset was partitioned into three distinct subsets: a training set consisting of 75% of the data, and a testing set with the remaining 25%.

#### 3.1.3 Data Preprocessing

To prepare the data for model input, all images were resized to a uniform dimension. A crucial preprocessing step was the normalization of pixel values; all images were scaled from their original [0, 255] integer range to a [0, 1] floating‐point range by applying a rescale factor of 1/255. Additionally, data augmentation was applied using the same ImageDataGenerator to simulate real‐world lighting conditions. Specifically, brightness was randomly adjusted between 80% and 120% of the original value. This helped the model learn to identify roast levels under varied lighting, improving generalization to unseen data.

#### 3.1.4 Model Selection

The selection of models for this study was driven by a strategy to benchmark a high‐performance deep learning architecture against a diverse set of powerful, classical ML paradigms.

Xception was chosen as the primary deep learning model due to its well‐established, state‐of‐the‐art performance on complex image classification tasks. Its sophisticated architecture makes it an ideal candidate for establishing a high‐accuracy benchmark for this specific problem.

To provide a robust comparison, we selected three classical ML models, each representing a different and effective classification philosophy: SVM for its strength in margin‐maximization, RF as a powerful bagging‐based ensemble method, and AdaBoost as a representative boosting algorithm. This selection ensures our deep learning model is benchmarked against a wide range of proven techniques. While computationally efficient, lightweight models like MobileNetV3 or EfficientNet‐lite exist, they were not included as the primary objective of this study was to determine the maximum achievable classification accuracy rather than to optimize for deployment on resource‐constrained devices.

#### 3.1.5 Model Training

The dataset was partitioned into training, validation, and testing sets using a two‐step process. First, 75% of the entire dataset was allocated for the training set. The remaining 25% was set aside as a temporary hold‐out pool. This hold‐out pool was then randomly partitioned to create the final validation and test sets. Specifically, 40% of the hold‐out pool was used for validation, and the remaining 60% was used for testing. This procedure resulted in a final data distribution where 75% of the total data was used for training, 10% for validation, and 15% for testing. A random_state was used during the second split to ensure reproducibility.

This split was chosen to align with common practices in ML and to suit the needs of our experimental setup. Allocating 75% of the data for training provides a substantial number of samples for the model to learn the underlying patterns effectively. The 10% validation set offers a dedicated, sufficiently large partition for unbiased monitoring of the training process, enabling reliable hyperparameter tuning and the implementation of early stopping. Finally, the remaining 15% is reserved as a completely unseen test set, providing a robust basis for the final, objective evaluation of the model's generalization performance.

#### 3.1.6 Model Evaluation

The performance of each trained model was rigorously evaluated on the test set using a suite of standard classification metrics. Overall performance was measured by accuracy, the ratio of correctly classified instances to the total number of instances. To gain deeper insight into per‐class performance, we calculated precision (the ability of the model to avoid labeling a negative sample as positive) and recall (the ability of the model to find all the positive samples).

The F1‐score, representing the harmonic mean of precision and recall, was then used to provide a single, balanced measure of a model's performance for each class. These metrics are computed as:

(1) $$\mathrm{Accuracy}\;=\frac{TP+FP}{TP+FP+TN+FN}$$

(2) $$\mathrm{Precision}=\frac{TP}{TP+FP}$$

(3) $$\mathrm{Recall}=\frac{TP}{TP+FN}$$

(4) $$F\;1_{\mathrm{score}}=\frac{2TP}{2TP+FP+FN}$$

where TP, TN, FP, and FN are true positive, true negative, false positive, and false negative samples, respectively.

Finally, a confusion matrix was generated for each model to provide a qualitative analysis of the error patterns, visualizing which roast levels were most frequently confused with one another. This comprehensive set of metrics facilitates a nuanced comparison of the models beyond simple accuracy.

### 3.2 Model Architecture

We developed two models: one using Google Colab with TensorFlow/Keras, based on the pre‐trained Xception model, and another using Orange software, testing models such as SVM, RF, and AdaBoost.

#### 3.2.1 CNN

**FIGURE 4:**
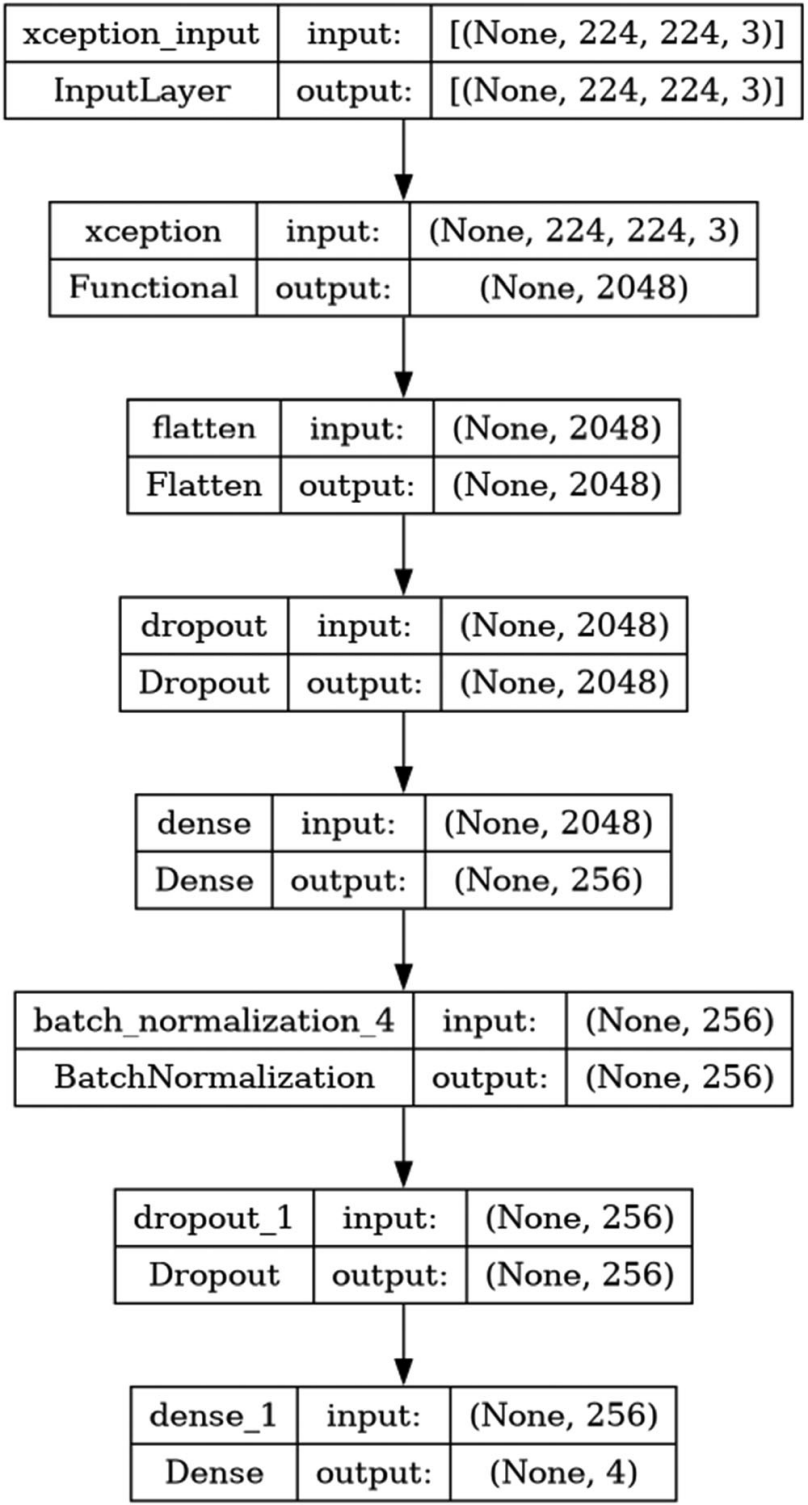
CNN model proposed using TensorFlow/Keras, based on the pre‐trained Xception model.

The core of our classification pipeline is a CNN built using a transfer learning approach with the TensorFlow Keras API, shown in Figure 4. The architecture utilizes the **X**ception model as its convolutional base, which was pre‐trained on the ImageNet dataset. The base model was instantiated with pooling = “max,” which applies global max pooling to the output of the convolutional layers. Its weights, derived from ImageNet, were used as the starting point for feature extraction from our 224 × 224 pixel RGB input images.

A custom classifier head was added on top of the Xception base. The architecture is defined as a Sequential model where the output from the base model is processed by the following sequence of layers:

1. A flatten layer to ensure the input to the dense layers is one‐dimensional.
2. A dropout layer with a rate of 0.3 (30%) for regularization.
3. A dense (fully‐connected) layer with 256 neurons using the ReLU activation function.
4. A batch normalization layer to stabilize and accelerate the training process.
5. A final dropout layer with a rate of 0.25 (25%) to further prevent overfitting.
6. An output dense layer with 4 neurons (one for each roast class), using a softmax activation function to produce class probabilities.

The entire model, including the layers of the Xception base, was made trainable in a fine‐tuning strategy.

For training, the model was compiled using the Adamax optimizer with a learning rate of 0.001. The categorical cross‐entropy loss function was employed, and model performance was monitored using the accuracy metric.

The primary CNN model was trained for a maximum of 20 epochs. To ensure efficiency and prevent overfitting, we implemented an EarlyStopping mechanism that monitored the validation loss (val_loss). This mechanism was configured with a patience of 3, meaning training would halt automatically if the validation loss did not improve for three consecutive epochs. This strategy allows the model to converge optimally without unnecessary training time.

#### 3.2.2 ML Methods: RF, SVM, and AdaBoost for Classification Tasks

To provide a comparative benchmark for our primary CNN model, we evaluated the performance of three classical ML algorithms: RF, SVM, and AdaBoost. These models were selected to represent diverse and powerful classification strategies: ensemble learning RF, margin maximization SVM, and boosting AdaBoost.

Since these algorithms operate on 1D feature vectors rather than raw image data, a feature extraction step was necessary. For this, we employed a pretrained InceptionV3 model as a feature extractor. Each image in our dataset was passed through the InceptionV3 architecture to generate a high‐dimensional feature embedding. This process leverages the rich visual representations learned by InceptionV3 on large‐scale datasets to create a compact and informative vector for each image, effectively reducing dimensionality while preserving critical patterns.

**FIGURE 5:**
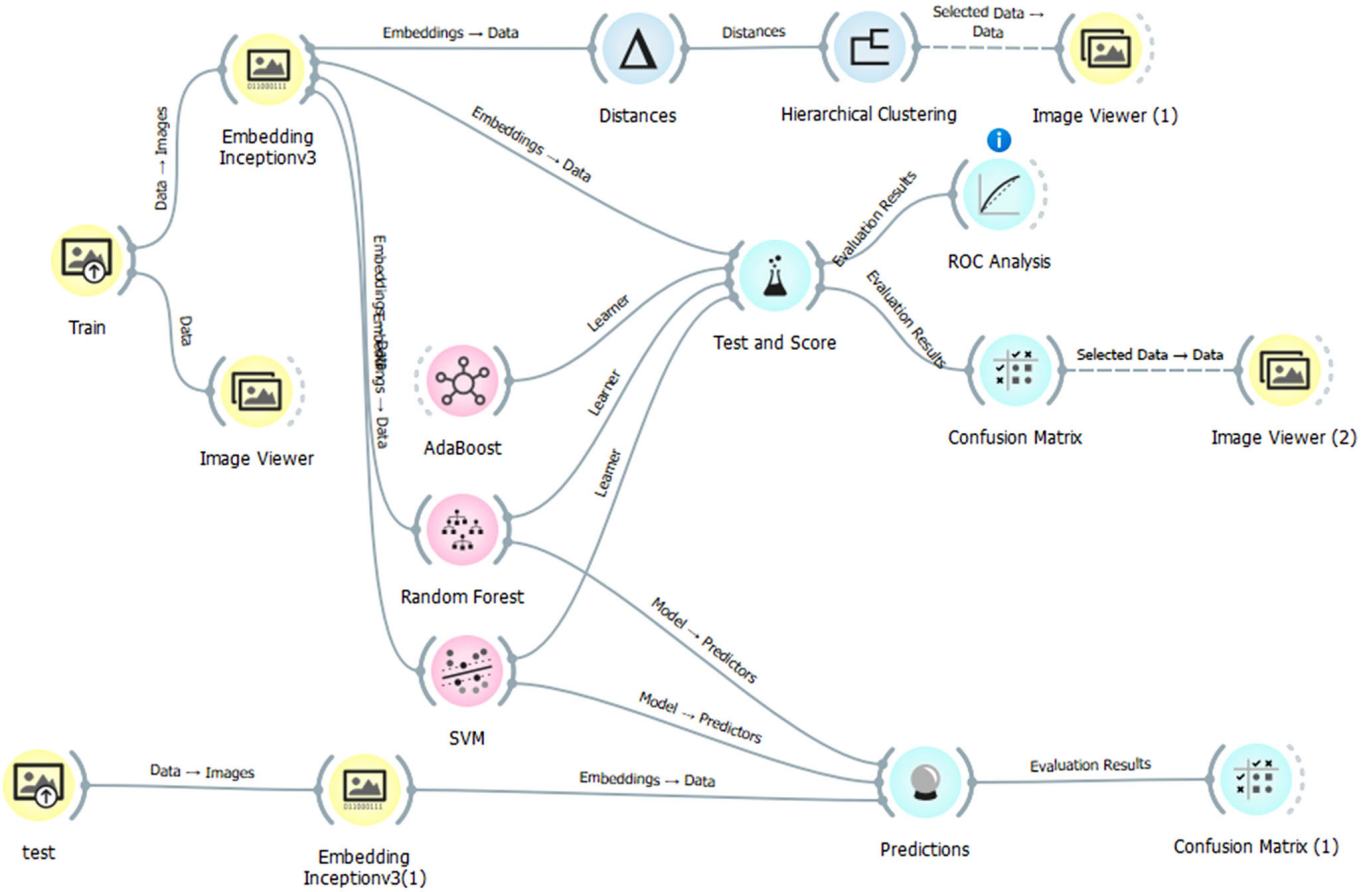
Classification model of roast levels using Orange software.

Using these embeddings as input, the RF, SVM, and AdaBoost models were trained and evaluated within the Orange Data Mining environment (see Figure 5). Performance was assessed using accuracy, precision, recall, F1‐score, and an analysis of the confusion matrix for each model, allowing for a comprehensive comparison against our deep learning approach.

## 4 Results

The complete code repository for this study is publicly available at (Garcia Rivas 2025). This repository includes the full implementation of our CNN and the workflow for the Orange Data Mining models, providing all the necessary resources to reproduce our findings and support further research.

### 4.1 CNN

**FIGURE 6:**
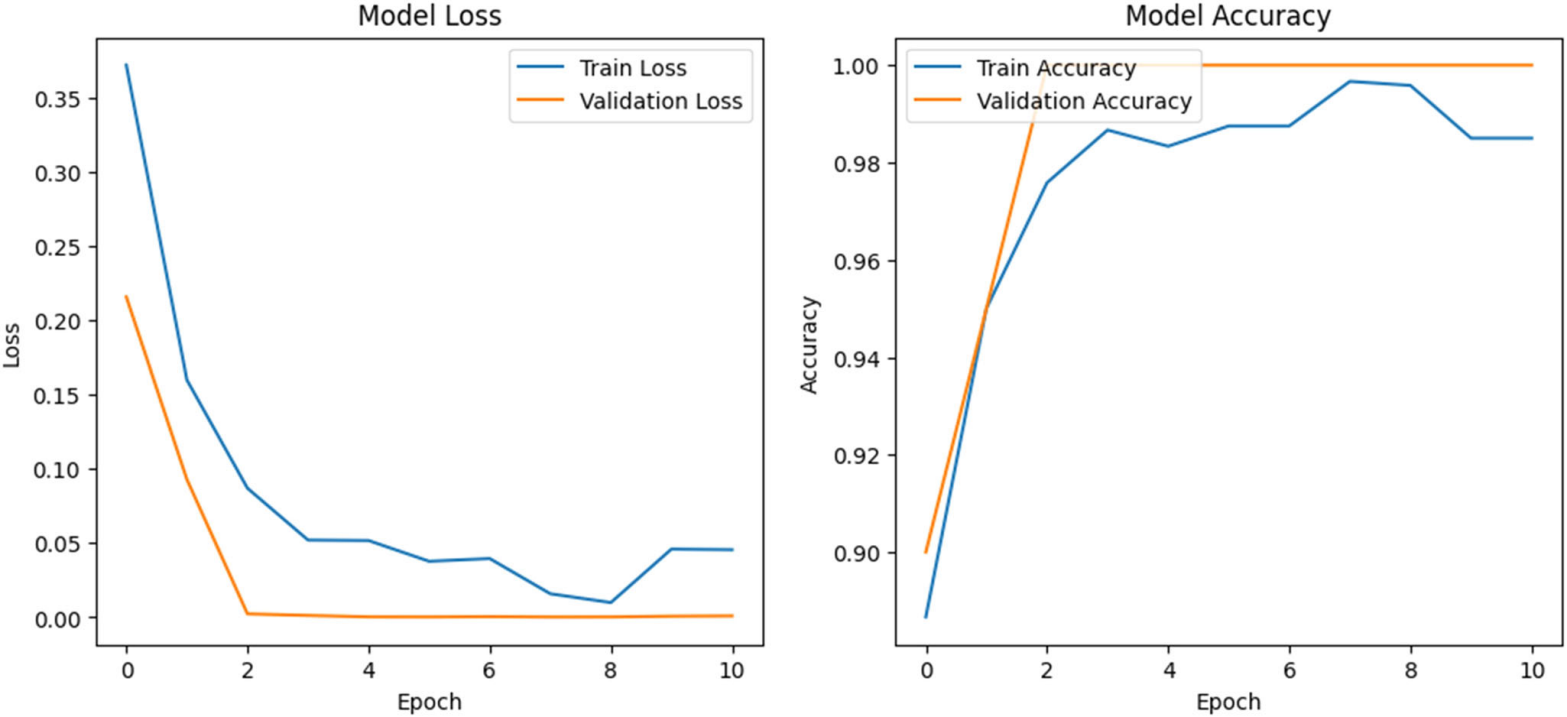
Training and validation accuracy and loss over epochs.

The CNN model, utilizing the fine‐tuned Xception architecture, achieved outstanding performance on the unseen test set. As shown by the training history in Figure 6, the model converged in just 10 epochs due to the early stopping mechanism, reaching a final validation accuracy of 100%.

Upon evaluation with the test set, the model maintained this perfect performance, achieving 100% for all key metrics: accuracy, precision, recall, and F1‐score. This indicates that the model was able to correctly classify every sample in the test set without error.

**FIGURE 7:**
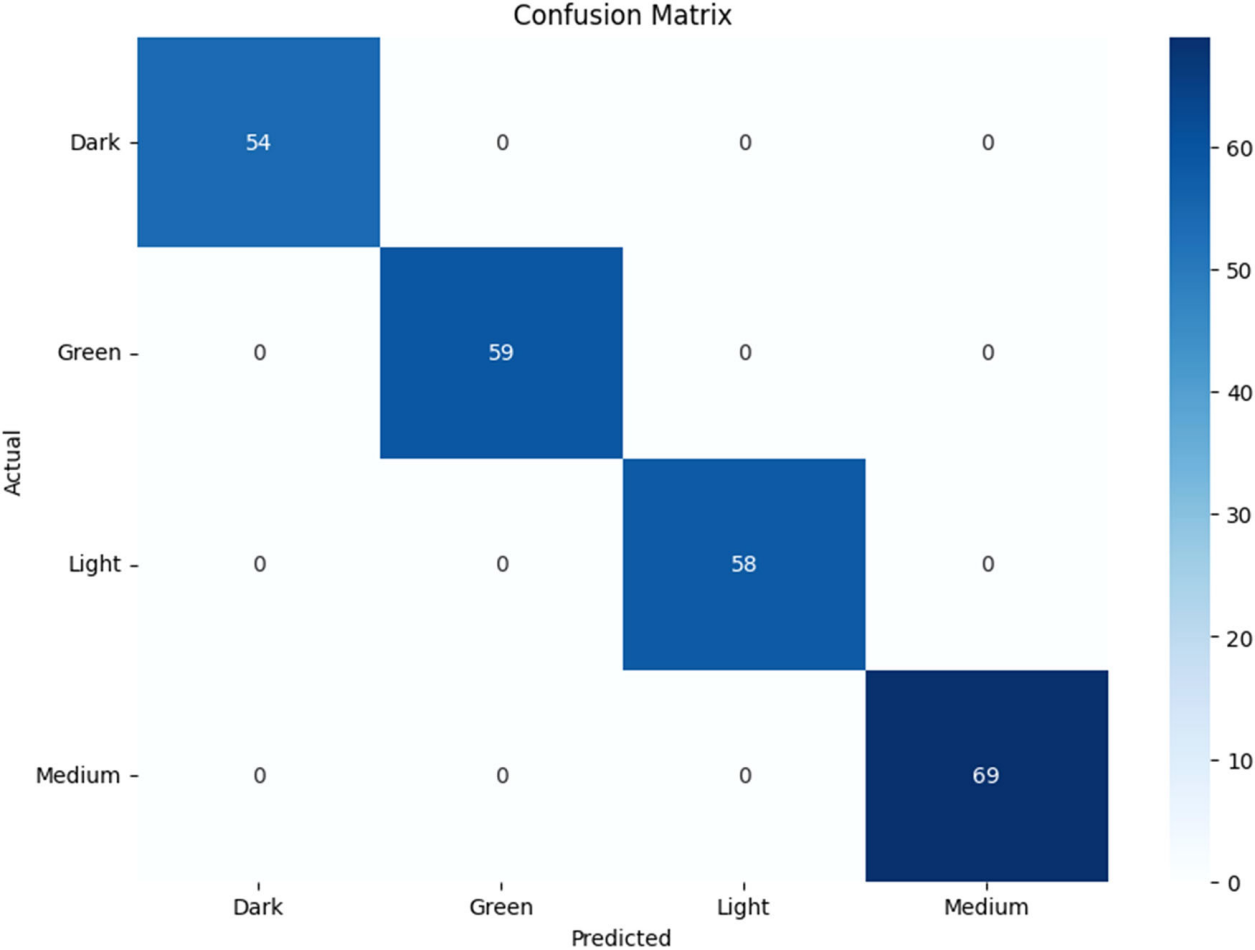
Confusion matrix of the CNN model.

**FIGURE 8:**
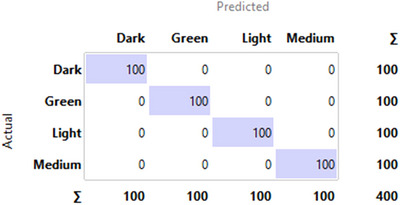
Confusion matrix of the SVM model generated in Orange Data Mining.

The confusion matrix for the CNN model, presented in Figure 7, visually confirms this result, showing a perfect diagonal with zero misclassifications. For comparison, the confusion matrix for the baseline SVM model is shown in Figure 8, illustrating the superior performance of the deep learning approach.

### 4.2 ML Methods: RF, SVM, and AdaBoost for Classification Tasks

**FIGURE 9:**
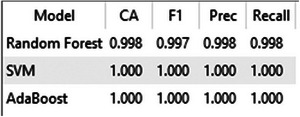
Performance metrics of SVM, RF, and AdaBoost models evaluated using Orange Data Mining.

The performance of the baseline ML models (AdaBoost, RF, and SVM) was evaluated using the InceptionV3 feature embeddings. A summary of the performance metrics for each model is presented in Figure 9.

Notably, both the RF and SVM models achieved perfect classification results, reaching 100% for all evaluated metrics, including accuracy, precision, recall, and F1‐score. The confusion matrix for the SVM model, shown in Figure 8, serves as a representative example of this flawless performance, displaying a perfect diagonal with zero misclassifications. The AdaBoost model achieved a slightly lower, yet still high, performance in comparison.

## 5 Discussion

The classification of coffee bean roast levels using CNNs is a growing area of research underpinned by foundational work in related fields. Studies utilizing computer vision to monitor the roasting process have demonstrated its effectiveness in identifying changes in properties across different roast levels (Bagdonaite and Murkovic 2018; Summa et al. 2007). Building on this foundation, recent advancements have leveraged CNNs to classify roast levels accurately. For instance, research by (Metha et al. 2024; Arboleda et al. 2019) applied CNN architectures to coffee bean images, achieving significant accuracy improvements over traditional methods. Specifically, the CNN‐based models reached accuracies as high as 100%, as illustrated in Figure 5, further showcasing the robustness of deep learning in this domain.

The results of this study demonstrate that deep learning models, particularly a fine‐tuned Xception CNN, can classify coffee roast levels with exceptionally high accuracy. Our primary model, as well as baseline RF and SVM models fed with InceptionV3 embeddings, all achieved 100% accuracy on the held‐out test set.

When compared to the 97.22% accuracy achieved by (Arboleda et al. 2019), the primary difference is architectural. Their use of a simple ANN with raw RGB values as input fails to capture the crucial spatial relationships within the image. Our CNN‐based approach, by contrast, is specifically designed to learn hierarchical spatial features, such as the subtle textures, edges, and color patterns that distinguish roast levels, providing a fundamentally more powerful method for image analysis.

Furthermore, our choice of the Xception architecture offers distinct advantages over the model used by (Metha et al. 2024), who achieved 94.79% with MobileNetV2. While MobileNetV2 is a highly effective model, it is explicitly designed and optimized for computational efficiency on edge devices, sacrificing some accuracy for speed and a smaller footprint. Xception, on the other hand, is a larger, more powerful architecture designed to maximize accuracy. Its use of depthwise separable convolutions represents a more modern and effective design than older architectures like VGG19, allowing for superior feature representation.

A central finding of this study was the 100% performance achieved by the primary CNN model on the held‐out test set. While such a result could initially raise concerns about overfitting, we validated the model's robustness and generalization capability through extensive k‐fold cross‐validation. A five‐fold cross‐validation resulted in a mean accuracy of 99.58% (± 0.36%), and a 10‐fold cross‐validation yielded a mean accuracy of 98.92% (± 1.5%).

The consistency of these high scores across numerous data folds demonstrates that the model's performance is not an artifact of a single favorable train‐test split. Instead, it reflects the model's strong ability to learn the visually distinct features of the roast levels present in this high‐quality dataset. This robust validation is crucial in supporting the claim that a fine‐tuned Xception architecture can, under these conditions, serve as a near‐perfect classifier.

An interesting finding was the perfect performance of the RF and SVM models. This suggests that the features extracted by the pre‐trained InceptionV3 model were highly separable, effectively transforming a complex image classification problem into a straightforward one for traditional classifiers. This highlights the power of transfer learning as a feature extraction technique. However, the end‐to‐end CNN approach remains advantageous as it combines feature extraction and classification into a single, optimized process, eliminating the need for a multi‐step pipeline.

A critical point of discussion is the 100% performance achieved by our fine‐tuned CNN and the baseline models using its embeddings. While the k‐fold cross‐validation confirms the model's robustness and low variance, such perfect scores merit a deeper, more critical analysis.

The primary reason for this exceptional performance is likely a confluence of two factors: the power of the transfer learning model and the nature of the dataset itself. The Xception architecture, pretrained on ImageNet, is exceptionally adept at discerning subtle visual features. When applied to a high‐quality, well‐controlled dataset where the four roast levels have distinct and consistent visual characteristics (color, texture, uniformity), the model can learn a near‐perfect decision boundary. This result serves as a powerful proof‐of‐concept, demonstrating the upper limit of performance achievable under ideal conditions.

However, these “ideal conditions” also represent the study's main limitation and demand a critical perspective. The dataset, while clean, was sourced from a single provider and photographed under controlled settings. Consequently, the model was not exposed to the full spectrum of real‐world variability, such as:

- Inter‐varietal differences: Beans from different varietals or origins that are roasted to the same level might exhibit different visual properties.
- Processing variations: Natural vs. washed processing can affect the final appearance of the bean.
- Roast defects: The dataset lacks images with common roast defects like scorching or tipping, which could confuse a classifier.
- Environmental noise: Uncontrolled lighting, shadows, and backgrounds present in a real production environment.

Therefore, while our model perfectly solved the problem presented by this specific dataset, it would be premature to claim it would achieve the same performance in a real‐world, industrial setting. The high accuracy should be interpreted as establishing a strong performance benchmark, rather than the final solution. The key challenge for future work is not to improve upon the 100% score, but to maintain a high level of accuracy when deploying the model against more diverse, noisy, and challenging datasets that better reflect the complexities of the coffee industry.

These models were conducted using the following hardware configuration: Intel Core i7 13th‐generation processor, Intel Iris Xe Graphics, 16 GB DDR4‐3200 MHz RAM, 512 GB SSD, and a P100 GPU for accelerated computation.

## 6 Future work

The achievement of near‐perfect accuracy has significant practical implications. A reliable, automated roast level classifier could be a valuable tool for quality control in the coffee industry, from small cooperatives to large‐scale roasting facilities, including here in El Salvador. It offers a method for ensuring consistency and adherence to quality standards that is objective, fast, and scalable.

However, this study has several limitations that open avenues for future research. First, the dataset, while clean, was sourced from a single provider and featured specific coffee varietals. Future work should validate the model's performance on a more diverse dataset encompassing different varietals, processing methods, and origins. Second, while imaging conditions were varied, testing the model in a true production environment with uncontrolled lighting and backgrounds is a critical next step. Finally, the scope of this work was limited to roast level; expanding the model to identify common roast defects (e.g., tipping, scorching) would dramatically increase its practical utility.

## 7 Conclusion

In conclusion, this study successfully demonstrates that a fine‐tuned CNN can serve as a highly accurate and robust tool for coffee roast classification. While traditional models also perform well with high‐quality features, the integrated CNN approach represents a more powerful and streamlined solution for complex visual analysis tasks in the coffee industry.

**Nomenclature**
ANN: Artificial Neural Network
BPNM: back‐propagation neural network
CNNs: Convolutional Neural Networks
DBN: Deep Belief Network
ICA: Imperialist Competitive Algorithm
KNN: k‐Nearest Neural Network
ML: Machine Learning
MLP: Multi‐Layer Perceptron
NNI: Neural Network Intensity
PCA: Principal Component Analysis
PLS‐DA: Partial Least Squares Discriminant Analysis
PNN: Probabilistic Neural Network
SVM: Support Vector Machine

## Author Contributions

**René Ernesto García Rivas**: conceptualization, investigation, methodology, writing – original draft, software, data curation. **Pedro Luiz Lima Bertarini**: validation, writing – review and editing, supervision, formal analysis. **Henrique Fernandes**: funding acquisition, methodology, validation, visualization, writing – review and editing, project administration, supervision.

## Conflicts of Interest

The authors declare no conflicts of interest.

## Data Availability

https://github.com/renerivas/coffee‐roast.
